# Tailored Intranasal Albumin Caged Selegiline-α Synuclein siRNA Liposome with Improved Efficiency in Parkinson’s Model

**DOI:** 10.3390/pharmaceutics17020243

**Published:** 2025-02-12

**Authors:** Ahmed A. Katamesh, Hend Mohamed Abdel-Bar, Mohammed Khaled Bin Break, Shimaa M. Hassoun, Gehad Mohammed Subaiea, Amr Radwan, Hadel A. Abo El-Enin

**Affiliations:** 1 Department of Pharmaceutics, College of Pharmacy, University of Ha’il, Ha’il 81442, Saudi Arabia; 2 Department of Pharmaceutics, Faculty of Pharmacy, University of Sadat City, Menoufia P.O. Box 32897, Egypt; 3 Department of Pharmaceutical Chemistry, College of Pharmacy, University of Ha’il, Ha’il 81442, Saudi Arabia; m.binbreak@uoh.edu.sa; 4 Medical and Diagnostic Research Centre, University of Ha’il, Ha’il 55473, Saudi Arabia; 5 Department of Pharmacology, College of Pharmacy, University of Ha’il, Ha’il 81442, Saudi Arabia; s.hassoun@uoh.edu.sa (S.M.H.); g.subaiea@uoh.edu.sa (G.M.S.); 6 Research Department, Academy of Scientific Research and Technology, Cairo 11694, Egypt; radwan.amro@gmail.com; 7 Egyptian Center for Innovation and Technology Development, Cairo 11512, Egypt; 8 Department of Pharmaceutics, Egyptian Drug Authority, Giza 12511, Egypt; hadelaboenin@outlook.com

**Keywords:** neurodegenerative disorder, siRNA delivery, dopamine, blood-brain barrier, pharmacokinetics

## Abstract

**Background/Objectives:** Parkinson’s disease (PD) is a progressive neuro-degenerative disorder characterized by α-synuclein aggregation, which promotes neuronal death and accelerates neurodegeneration. Small interfering RNA (siRNA) can reduce α-synuclein levels, but its therapeutic potential is limited by poor stability and delivery challenges. Similarly, Selegiline (Sel), a monoamine oxidase-B (MAO-B) inhibitor, has low bioavailability, restricting its effectiveness. This study aims to develop an intranasal (IN) albumin-coated liposomal system (C-Lip_Sel-siSNCA2_) for the co-delivery of Sel and α-synuclein-targeting siRNA (siSNCA2) to enhance brain targeting and therapeutic efficacy. **Methods:** Liposomes were prepared using the ethanol injection method and optimized via D-optimal design for size, charge, and encapsulation efficiency (EE%). The optimized formulation was coated with human serum albumin (HSA) and characterized for stability, cellular uptake, and gene silencing. In vivo pharmacokinetics and pharmacodynamics were assessed in a rotenone-induced PD rat model to evaluate the motor function, biochemical markers, and brain-targeting efficiency. **Results:** Optimized liposomes had a particle size of 113.5 ± 6.8 nm, zeta potential of 6.2 ± 0.8 mV, and high EE% (Sel: 92.35%; siRNA: 78.66%). Albumin coating increased size to 136.5 ± 10.3 nm and shifted zeta potential to −13.5 ± 1.4 mV, enhancing stability and targeting. IN administration achieved a 3-fold increase in brain area under the concentration-time curve (AUC) versus intravenous delivery. In PD rats, C-Lip_Sel-siSNCA2_ improved motor and non-motor functions, restored dopamine levels, enhanced catalase activity, and reduced MAO-B levels, mitigating dopamine degradation and α-synuclein aggregation. **Conclusions:** This non-invasive, dual-action nanoplatform offers a targeted therapy for PD, combining siRNA gene silencing and MAO-B inhibition, with the potential for clinical translation in neurodegenerative diseases.

## 1. Introduction

Parkinson’s disease (PD) is a chronic and progressive neurodegenerative disorder that presents a significant public health challenge, particularly as the global population ages. The prevalence of PD is projected to increase dramatically, potentially affecting over 12 million people by 2040 [1]. Clinically, PD is characterized by a range of motor symptoms, including tremors, rigidity, bradykinesia, and postural instability, which primarily result from the degeneration of dopaminergic neurons in the substantia nigra pars compacta (SNpC). This neuronal loss disrupts dopaminergic pathways, critically impairing motor control and leading to the hallmark symptoms of PD [2,3]. Beyond motor deficits, patients also suffer from non-motor symptoms such as cognitive impairment, mood disorders, and autonomic dysfunction, further complicating the disease burden [4,5].

A critical pathological hallmark of PD is the accumulation of misfolded α-synuclein protein, which aggregates into toxic fibrils, forming Lewy bodies and Lewy neurites. These aggregates not only contribute to neuronal death but also propagate to neighboring cells, exacerbating the neurodegenerative process [6]. Given the central role of α-synuclein in PD pathogenesis, therapeutic strategies aimed at reducing α-synuclein expression or preventing its aggregation have become a focal point in PD research [7]. Sela and co-workers developed brain-targeted liposomes encapsulating SynO4, an anti-α-synuclein monoclonal antibody designed to inhibit α-synuclein aggregation. Their findings demonstrated that this targeted approach significantly enhanced neuronal survival, improved motor coordination, and boosted cognitive function in PD mouse models [8].

Among innovative therapeutic strategies, RNA interference (RNAi) has shown substantial promise, particularly using small interfering RNA (siRNA) to silence the SNCA gene [9], which encodes α-synuclein. By specifically targeting SNCA mRNA, siRNA can effectively reduce α-synuclein levels, potentially mitigating its neurotoxic effects and slowing disease progression [10,11]. However, the clinical application of siRNA faces significant hurdles, primarily due to the challenges associated with its delivery. The blood-brain barrier (BBB), a highly selective permeability barrier, poses a significant obstacle to the delivery of siRNA to the central nervous system (CNS) [12]. Moreover, siRNA molecules are inherently unstable in the bloodstream, susceptible to rapid degradation by nucleases, exhibit poor cellular uptake, and can trigger immune responses, complicating their therapeutic use further [13,14].

Various approaches have been explored to enhance siRNA delivery, including the use of viral vectors, nanoparticles, and lipid-based carriers [15]. While viral vectors are effective, they raise concerns regarding immunogenicity and insertional mutagenesis. Nanoparticles, including gold and polymeric varieties, have shown potential, but their biocompatibility and long-term safety remain under investigation.

Nanobiotechnology has revolutionized drug delivery by enabling precise, targeted therapies that overcome traditional pharmacological barriers [16]. In neurological disorders, where the BBB limits therapeutic access to CNS, nanocarrier-based strategies have emerged as promising delivery platforms [12]. Among nanocarrier-based systems, liposomes, polymeric nanoparticles, solid lipid nanoparticles, dendrimers, and exosomes have demonstrated significant potential in facilitating drug transport across biological barriers, including the BBB. These nanoscale systems leverage unique physicochemical properties, such as high surface area, tunable surface charge, and functionalization capabilities, to enhance drug stability and bio-distribution [17].

Liposomes, nanoscale lipid carriers that mimic biological membranes, have emerged as particularly promising vehicles for different therapeutics and siRNA delivery due to their biocompatibility, ability to encapsulate nucleic acids, and potential for surface modification to achieve targeted delivery [18,19]. Liposomes can cross the BBB through diverse transport mechanisms, including adsorptive-mediated transport, receptor-mediated transport, and carrier-mediated transport [20]. Unlike conventional drug formulations, liposomal strategies incorporate physiological interactions, allowing the drug carrier to influence its own distribution profile, thereby enhancing CNS penetration. Different studies have demonstrated the ability of liposomes to improve the therapeutic outcome of different therapeutics in CNS disorders such as Alzheimer’s disease, brain viral infection, brain cancer, and PD [21,22,23,24]. Moreover, liposomes can protect siRNA from enzymatic degradation, enhance its pharmacokinetic profile, and facilitate its transport across cellular membranes, thereby improving its therapeutic efficacy in PD. Despite these advantages, liposomes face several challenges, such as instability, short circulation half-life due to rapid clearance by the reticuloendothelial system (RES), and drug leakage. These issues underscore the need for ongoing research to enhance the stability, efficacy, and targeted delivery capabilities of liposomes [25,26].

To address these challenges, coating liposomes with albumin has emerged as a promising strategy. Albumin, a naturally occurring and highly biocompatible protein, significantly enhances the circulation time of liposomal formulations by reducing their recognition and clearance by RES [25]. Additionally, albumin facilitates the penetration of the BBB through receptor-mediated transcytosis, interacting with specific receptors on brain endothelial cells. This mechanism not only ensures that therapeutic agents encapsulated within the liposomes are effectively delivered to the brain but also leverages albumin’s natural pathways to enhance the precision and efficiency of drug delivery in PD treatment. Albumin-coated liposomes can also be engineered for controlled drug release, ensuring sustained therapeutic levels over time while minimizing systemic toxicity and side effects [26].

Selegiline (Sel), a selective monoamine oxidase B (MAO-B) inhibitor, has long been a mainstay in the pharmacological management of PD. By inhibiting MAO-B, Sel prevents the breakdown of dopamine, thereby increasing its availability in the brain and alleviating motor symptoms associated with dopaminergic neuron loss) [27]. However, the therapeutic use of Sel is limited by its pharmacokinetic profile, as oral administration is hampered by poor bioavailability due to extensive first-pass metabolism, and its ability to cross the BBB is limited) [28]. These limitations have spurred interest in developing alternative delivery methods to enhance CNS penetration and improve the therapeutic outcomes of Sel [29].

Recent studies have demonstrated that nanocarrier systems, such as polymeric nanoparticles and lipid-based systems, can enhance the bioavailability and CNS targeting of Sel [30,31]. For instance, co-delivery of Sel with neuroprotective peptides using nanocarriers has shown promising results in PD models, suggesting that such strategies can enhance neuroprotection and improve motor function [32]. However, the combined delivery of Sel and siRNA targeting α-synuclein has not been extensively studied, despite the potential for synergistic effects that could more effectively slow or halt disease progression.

This study focuses on the development and evaluation of a novel intranasal (IN) delivery system utilizing albumin-coated liposomes for the co-delivery of Sel and α-synuclein-targeting siRNA. The IN route offers a compelling method for delivering therapeutic agents directly to the CNS [33,34,35]. It bypasses the BBB by utilizing the olfactory and trigeminal neural pathways, enabling efficient and direct drug delivery to the brain [36,37,38]. The non-invasive nature of IN delivery, coupled with its potential to minimize systemic side effects and enhance CNS bioavailability, makes it a promising strategy for improving the efficacy and safety of treatments in PD [39,40]. Using a D-optimal design approach, the liposomal formulation was optimized to achieve minimal particle size, optimal zeta potential, and high encapsulation efficiency (EE%) for both Sel and siRNA. The optimized formulation was characterized by its physicochemical properties, in vitro release profile, and in vivo pharmacokinetics and pharmacodynamics in a rotenone-induced PD rat model. Our findings demonstrate that the optimized platform significantly improves brain delivery of Sel and siRNA, restores dopamine levels, and mitigates motor deficits in the PD rat model, highlighting its potential as a novel therapeutic strategy for PD. This study lays the groundwork for future biological and toxicological investigations of this innovative IN delivery system. By enhancing the stability and brain-targeting efficiency of the formulation, albumin-coated liposomes represent a significant advancement in the field of nanomedicine for neurodegenerative diseases, particularly in the treatment of PD. The success of this platform may open new avenues for the development of multifunctional nanocarriers capable of delivering combination therapies directly to the brain, offering hope for more effective treatment strategies in the fight against PD.

## 2. Materials and Methods

### 2.1. Materials

Selegiline (SeL), Cholesterol (CH), citric acid, sodium citrate, dimethylsulphoxide (DMSO), absolute ethanol, Triton X-100, Hydrogen peroxide, and human serum albumin (HSA) were purchased from Sigma Aldrich (St. Louis, MO, USA). 3-(4,5-Dimethylthiazol-2-yl)-2,5-diphenyltetrazolium bromide (MTT), sodium thiopental and rotenone were purchased from Thermo Scientific™ (Loughborough, UK). Minimum Essential Medium (MEM), trypsin-EDTA, fetal Bovine Serum (FBS), penicillin/ streptomycin, L-Glutamine, and phosphate buffer saline (PBS, pH 7.4) were purchased from Gibco, Invitrogen (Milton Keynes, UK). 1, 2-distearoyl-snglycero-3-phosphocholine(DSPC),1, 2-dioleoyl-3-trimethylammonium-propane(DOTAP), N-palmitoyl-sphingosine-1-[succinyl (methoxypolyethylene glycol) 2000] (C16-PEG2000 Ceramide) were purchased from Avanti Polar Lipids (Alabaster, AL, USA). α-Synuclein siRNA (siSNCA_2_) and non-Targeting siRNA Control (siNEG) were purchased from Santa Cruz Biotechnology (Dallas, TX, USA). siAtto655 was obtained from Dharmacon (Cambridge, UK). Monoclonal α-Synuclein- Alexa Fluor^®^ 647 antibody (clone 2A7) and mouse IgG1- Alexa Fluor^®^ 647 isotype were purchased from Bio-Techne Ltd. (Abingdon, UK). SH-SY5Y human neuroblastoma (catalog number: CRL-2266™) and Calu-3 (catalog number: HTB-55™) cell lines were purchased from ATCC, USA (Manassas, VA, USA).

### 2.2. Preparation of Sel-siRNA Loaded Liposomes

Different Sel-siRNA-loaded liposomes (Lip_Sel-siNEG_) were prepared using the ethanol injection method as described elsewhere [41]. Briefly, different molar ratios (based on several preliminary experiments) of CH, DSPC, DOTAP, and C16-PEG2000 ceramide at a total lipid amount of 0.22 µm and Sel (5 mg) were dissolved in absolute ethanol (200 µL) at 60 °C. The aqueous phase composed of siNEG (1 nmole) was dissolved in citrate buffer pH 4 (20 mM, 300 µL) prepared using RNAse-free water at 60 °C was titrated with the alcoholic lipid mixture under vigorous mixing with vortex. The obtained formulae were incubated for 1 h at 40 °C. The unencapsulated Sel and siNEG were removed using ultrafiltration centrifugation (MWCO 100 K) at 14,000 rpm for 45 min. The collected Lip_Sel-siNEG_ was re-dispersed in PBS pH 7.4 (0.5 mL, RNAse-free water).

### 2.3. Optimization of Sel-siRNA Loaded Liposomes Using D-Optimal Design

Four factors, each at two levels of D-optimal design, were implemented to statistically optimize the preparation of different Lip_Sel-siNEG_ (design-expert^®^ 13, State-Ease Inc., Minneapolis, MN, USA). Table 1 shows the different molar ratios of the four used lipids (CH, DSPC, DOTAP, C16-PEG2000), where the total ratio is kept at 100%. The influence of lipids molar ratio on the obtained liposomes particle size, zeta potential as well as both Sel and siRNA EE% were assessed. Surface response and polynomial equations were created where the best fitting model, either linear, two-factor interactions (2FI), or quadratic model was selected based on R^2^ values (adjusted and predicted) and predicted residual error sum of squares (PRESS). The optimized formula was selected based on the lowest particle size and the maximum zeta potential, Sel, and siRNA EE%.

### 2.4. Preparation of HSA Coated Lip_Sel-siNEG_

HSA-coated Lip_Sel-siNEG_ were prepared by suspending the optimized formula in different concentrations of HSA dissolved in PBS pH 7.4 (100–1000 µg/mL), prepared in RNAse-free water, in a volume ratio of 1:1. Samples were incubated at 37 °C for 2 h with gentle stirring to allow the formation of the HSA coat [18,42]. The obtained HSA-coated liposomes (C-Lip_Sel-siNEG_) were purified by ultrafiltration centrifugation (MWCO 100K) at 14,000 rpm for 45 min at 4 °C. The collected liposomes were re-dispersed in PBS pH 7.4 (0.5 mL, RNAse-free water). siNEG was replaced by siAtto655 for cellular uptake study. Cytotoxicity, gene silencing, and therapeutic studies were conducted by replacing siNEG with siSNCA_2_.

### 2.5. In Vitro Characterization of the Different Sel-siRNA Loaded Formulae

#### 2.5.1. Determination of Particle Size and Zeta Potential

Particle size, and size distribution expressed as polydispersity index (PDI) of different Lip_Sel-siNEG_ and C-Lip_Sel-siNEG_ were measured using dynamic light scattering (DLS). Zeta potential was quantified using electrophoresis. Briefly, different formulations were diluted using deionized water (1:100 *v*/*v*) and transferred to disposable plain folded capillary Zeta cells. All the measurements represent the average of 20 runs, each run was completed in triplicate at 25 °C [43].

#### 2.5.2. Determination of Sel and siRNA Encapsulation Efficiency

Both Sel and siRNA EE% were quantified directly using previously validated HPLC method and RiboGreen assay, respectively. Briefly, free unencapsulated Sel and siNEG were separated using ultrafiltration centrifugation (MWCO 30KDa) at 14,000 rpm for 45 min at 4 °C. The obtained formulae were dissolved in PBS pH 7.4 containing Triton X-100 (10% *w*/*v*). The amount of Sel was consequently measured using HPLC method [44]. siRNA EE% was measured using Quant-iT RiboGreen assay (Invitrogen™, UK) according to the manufacturer’s protocol. The dissolved liposome samples were mixed with RiboGreen working solution in a ratio of 1:1. Samples were added to a black-96 well plate and fluorescence intensity was measured at 485 nm and 520 nm for excitation and emission, respectively, using microplate reader (FLUOstar^®^ Omega, Rotherham, UK). The EE% of both Sel and siRNA was calculated using the following equation:

(1) $$\mathrm{EE}\%=\frac{amount\;of\;either\;Sel\;or\;siRNA\;inside\;the\;liposome}{Initial\;amount\;of\;Sel\;or\;siRNA\;added}\times 100$$

#### 2.5.3. Determination of HSA Amount on the Surface of Coated Liposomes

The amount of HSA adsorbed on the surface of Lip_Sel-siNEG_ was measured by Bicinchoninic Acid (BCA) protein assay (Pierce™ BCA Protein Assay Kit, Thermo Scientific, UK) following the manufacturer’s protocol. C-Lip_Sel-siNEG_ was mixed with BCA reagents A and B for 30 min at 37 °C. The absorbance was measured at 562 nm in a microplate reader [45].

#### 2.5.4. In Vitro Serum Stability Study

The in vitro serum stability of the selected C-Lip_Sel-siNEG_ was assessed by recording the particle size, PDI, and zeta potential after incubation with 10% and 50% *v*/*v* fetal bovine serum (FBS) for 4, 24, and 48 h at 37 ± 0.5 °C. The measurements were made as described before.

#### 2.5.5. In Vitro Sel Release

The in vitro release of Sel from C-Lip_Sel-siNEG_ was conducted using dialysis technique. Briefly, an aliquot volume of C-Lip_Sel-siNEG_ (equivalent to 2 mg of Sel) was placed in a dialysis sac (MW cutoff 10 KDa). The tightly closed dialysis sac was suspended in a flask containing simulated nasal fluid (pH 7.4, 50 mL) at 35 °C, the reported nasal temperature, in a shaking water bath at 50 rpm. At predetermined time intervals, samples (0.5 mL) were withdrawn and replaced by fresh simulated nasal fluid. The drug in the samples was analyzed using the validated HPLC method and the percentage of Sel released was calculated.

#### 2.5.6. Transmission Electron Microscopy

Morphological architecture of C-Lip_Sel-siNEG_ was visualized using Transmission Electron Microscope (TEM, JEOL JEM1230, Tokyo, Japan) [46].

#### 2.5.7. In Vitro Cytotoxicity

Biocompatibility of the optimized C-Lip_Sel-siNEG_ was assessed on SH-SY5Y and Calu-3 cells using MTT assay. In brief, SH-SY5Y and Calu-3 cells were cultured individually in MEM media supplemented with FBS (10% *v*/*v*), L-glutamine (2 mM), and a mixture of penicillin and streptomycin (1% *v*/*v*) in 5% CO_2_ at 37 °C in T-75 tissue culture flask. SH-SY5Y cells were pretreated with rotenone (10 nM) for 5 days to induce α-synuclein overexpression. Afterwards, either Calu-3 or α-synuclein overexpressed SH-SY5Y cells that had grown exponentially and reached 80% confluency were harvested using 0.25% trypsin-EDTA. Consequently, cells were then seeded into 96-well plates at a density of 7k cells per well in MEM supplemented medium and cultured for 24 h. Cells were treated with C-Lip_Sel-siSNCA2_ with serial concentration of Sel equivalent to 0.01–100 µM for 48 h. Afterwards, culture media were aspirated and replaced with MTT solution (120 µL) then cells were incubated for 4 h at 37 °C and 5% CO_2_. The obtained formazon crystals were dissolved in DMSO (200 µL) and the optical density was measured at 570 nm using plate reader. Cell viability was expressed as the ratio between survived treated cells to the control untreated ones [47].

#### 2.5.8. Cellular Uptake Study

Cellular internalization of the optimized fluorescence-labeled siRNA was quantified in SH-SY5Y cells. Briefly, cells were seeded in a 24-well plate at a density of 50 k/well in MEM media for 24 h. Consequently, SH-SY5Y cells were treated with C-Lip_Sel-siAtto655_ at siRNA concentrations equivalent to 10, 20, and 30 nM for 4 and 24 h. Cells were washed with PBS (pH 7.4, ×3), harvested, and centrifuged at 513× *g* for 3 min at 4 °C. The collected cells were suspended in PBS (pH 7.4, 200 µL) and siRNA uptake was quantified using Flow cytometry (BD FACSCalibur™, BD Biosciences, San Jose, CA, USA) by measuring the mean fluorescence intensity (MFI) using the fluorescence FL4 detector at 10 k gated cells. The analysis was carried out using Flowjo software (Treestar) [48].

#### 2.5.9. In Vitro Gene Silencing

To assess the gene silencing potential, α-synuclein overexpressed SH-SY5Y cells were prepared using rotenone as described before. Consequently, cells were seeded in 24-well plates at density of 50 k/well for 24 h. Consequently, SH-SY5Y cells were incubated with C-Lip_Sel-siSNCA2_ at a concentration of 10, 20, and 30 nM for 48 h. Cells were washed with PBS (pH 7.4) two times and collected with trypsin-EDTA. Cells were stained with monoclonal α-Synuclein-Alexa Fluor^®^ 647 antibody. Consequently, α-Synuclein expression was quantified by measuring the fluorescence of 10k gated cells using FL4 detector (BD FACS Calibur flow cytometer, BD Biosciences). Analysis was performed using Flowjo software (Treestar). Transfection efficiency was calculated as the percentage of positive cells.

#### 2.5.10. In Vivo Pharmacokinetic Study

All animal experiments were conducted in compliance with the guidelines of the ethical committee, Faculty of Pharmacy, University of Sadat City (approval number RERC-FOP-USC-24-02-08). Adult male albino rats (200 g ± 10%) were randomly divided into two groups each of 48 animals. Animals were subjected to anesthesia using sodium thiopental via intraperitoneal injection (IP). Consequently, rats received Sel at a dose of 1 mg/kg either via IV injection through the tail vein or IN instillation of 10 µL of C-Lip_Sel-siSNCA2_ containing 0.75 mg/kg siSNCA2 in each nostril. At each time point, blood samples were collected from 6 animals by cardiac puncture on heparinized tube and then animals were sacrificed. Blood samples were centrifuged at 9000 rpm and the obtained plasma was stored at −80 °C until analysis. The whole brain was isolated, washed with saline, and homogenized at 10,000 rpm in ice-cold PBS (pH 7.4) for 5 min. The homogenate obtained was centrifuged at 9000 rpm for 15 min. Sel concentration in plasma and brain was quantified using LCMS/MS [49,50]. The maximum drug concentration (C_max_), time required to achieve C_max_ (T_max_), area under the concentration-time curve (AUC_0–480min_ and AUC_0–∞_), mean residence time (MRT), and elimination rate constant (Kel) in both plasma and brain were calculated. All pharmacokinetics data were calculated using the pharmacokinetic software PK Solver-Add Ins for Microsoft Excel 2007. Additionally, absolute bioavailability as well as the Direct-Transport Percentage (DTP%) and Drug-Targeting Efficiency percentage (DTE%) were calculated using the following equations [51]:

DTE = (AUC_brain_/AUC_plasma_) IN/(AUC_brain_/AUC_plasma_) IV × 100 (2)

DTP% = B_IN_ − BX/B_IN_ × 100 (3)

where B_X_ = B_IV_/P_IV_ × P_IN_

BX is the brain AUC_0–480min_ fraction contributed by systemic circulation, following IN administration; B_IV_ is the brain AUC_0–480min_ following IV administration; P_IV_ is the plasma AUC_0–480min_ following IV administration; B_IN_ is the brain AUC_0–480min_ following IN administration; P_IN_ is the plasma AUC_0–480min_ following IN administration.

#### 2.5.11. In Vivo Therapeutic Study in Parkinson’s Disorder Simulated Model

##### Stride Length Measurement

PD was simulated in rats by IP injection of rotenone (3 mg/kg/day) for 11 days [52,53]. On days 12 and 20 post-starting rotenone injection, rats received either IN C-Lip_Sel-siSNCA2_ or IV Sel solution. On day 30 of the experiment, stride length was recorded based on the spatial distance a paw travels during a stride. Animal forepaws were dipped in black non-toxic ink. The length of forelimb and hindlimb steps in the left and right limbs were measured while walking on a clean piece of paper. Stride length was defined as the distance between each step on the same side of the animal’s body, measured from the mid-digit toe of the first step to the heel of the second [54].

##### Paw Placement and Coverage

On day 30, the distance between the location of the behind paw and the previously placed contralateral front paw was measured during the same step cycle [55] while the area covered when the forelimbs or hindlimbs perform a stride was recorded as the coverage area [56].

##### Open Field Test

An open-field device with 16 sub-squares (40 × 40 × 30 cm; Accuscan Instruments, Columbus, OH, USA) was utilized to examine forelimb and hindlimb locomotor impairments in rats following 30 days of PD induction. Prior to the test, each rat was left in its home cage in the testing area for 2 h before being gently carried by the base of its tail to the center of the open field arena. The animal behavior was then studied for 60 min. To measure the rat’s ambulatory distance, the total number of squares crossed was recorded [57]. Additionally, one of the non-motor characteristics of PD that has been investigated is anxiety, which is indexed by the preference exploration of the open field’s periphery. The number of individuals crossing the center zone was noted [58].

##### Catalepsy Test

The catalepsy score was determined using the bar method, which involved measuring the time that rat consumed to remove its forepaws from the rod. The rats were rated on a five-point scale: 0 (0–5 s), 1 (6–15 s), 2 (16–25 s), 3 (26–35 s), 4 (36–60 s), and 5 (>60 s). The rat’s forepaws were gently put on a steel rod positioned 10 cm above the bench. The recording begins when cataleptic animals have been held in this position for at least 30 s (the adapting time) [59].

##### Determination of Dopamine Level and Catalase Activity

On day 30 of the experiment, rats were scarified and brain tissues were collected. Brain tissues were homogenized as previously described and the concentration of dopamine in the brain homogenate was assessed by HPLC-equipped with a fluorescence detector set at excitation/emission wavelengths of 280/315 nm as described elsewhere [60]. Catalase activity in brain homogenate was measured using colorimetric assay at 570 nm using hydrogen peroxide [61].

##### Measurement of MAO-B

The levels of MAO-B in brain homogenates expressed as ng/g tissue were quantified using ELISA kit (Abbexa, Cambridge, UK) as described by the manufacturers’ prescription [62].

#### 2.5.12. Statistical Analysis

All in vitro results are displayed as mean ± standard deviation (SD) of three replicates. In vivo study results were represented as the mean of six animals ± standard error (SE). Student’s *t*-test was applied to compare two variables while ANOVA test followed by Tukey HSD test was used for comparing different parameters between groups where *p* < 0.05 was considered significant.

## 3. Results and Discussion

### 3.1. Preparation and Optimization of Sel-siRNA Loaded Liposomes

**Figure 1:**
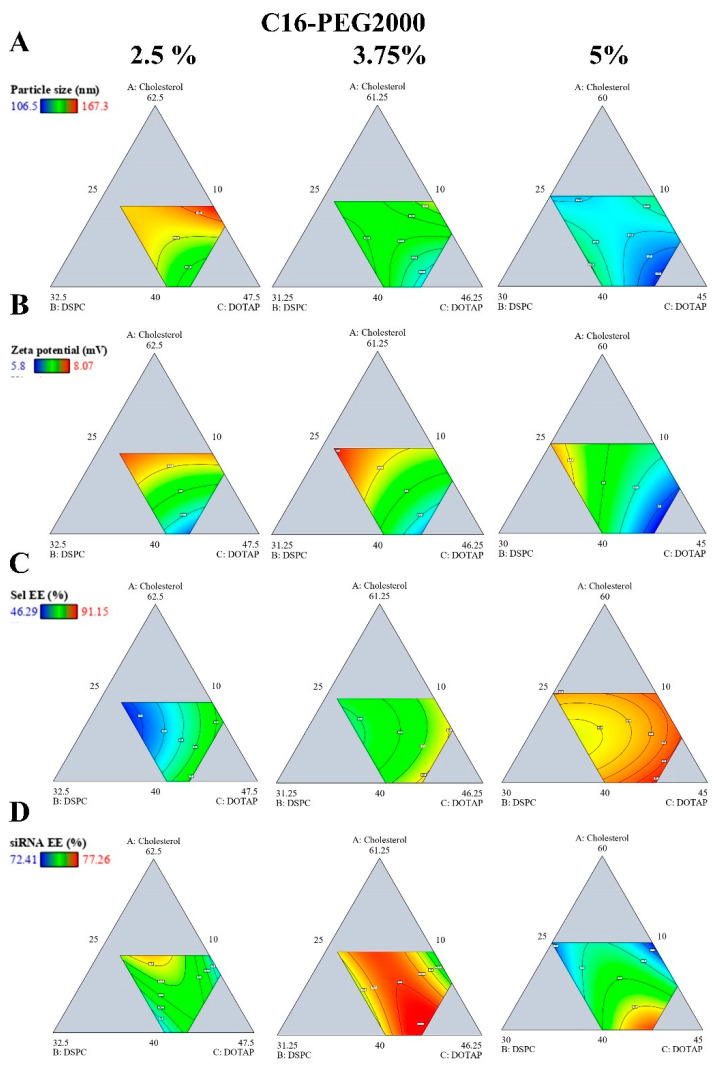
**The effect of lipid composition on particle size, zeta potential, Sel, and siRNA encapsulation efficiency.** Data obtained from a physicochemical characterization of the prepared liposomes were used to generate a predictive DoE model. Lipid interactions defined between CH (**A**), DSPC (**B**) and DOTAP (**C**) when C16-PEG2000 (**D**) was used at 2.5% (**left**), 3.75% (**middle**), 5% (**right**) on particle size (Y1) (**A**), zeta potential (Y2) (**B**), Sel EE% (Y3) (**C**), and siRNA EE% (Y4) (**D**) are presented as contour plots.

The study utilized a design of experiments (DoE) methodology to optimize the formulation of liposomes for effective loading of Sel and siRNA targeting α-synuclein. Various lipid compositions were trialed to determine optimal conditions that achieved minimal particle size and maximal drug and siRNA EE%. Table S1 shows the composition of the 20 fabricated Sel-siRNA loaded liposomes and the obtained responses namely, particle size, zeta potential, and EE% for both Sel and siRNA. The DoE approach was used to suggest the most fitting statistical model based on its high R2 and low PRESS values with a signal-to-noise ratio of more than four. Consequently, the quadratic model was selected as the best-fit statistical model for all responses investigated. The suggested model had ANOVA study was significant (*p* < 0.05) and lack of fit was not significant (*p* > 0.05). The lipid interactions defined between different formulation components were identified at three interaction points when the C16-PEG2000 was maintained at 2.5%, 3.75%, and 5% to study the effect on different responses which are presented as contour plots and 3D surface plots (Figure 1 and Figure S1).

The particle size of liposomes is a critical parameter that affects their distribution, cellular uptake, and overall therapeutic efficacy [63]. The particle size of the liposomes varied depending on the lipid molar composition from 106.5 ± 4.3 nm to 167.3 ± 5.6 nm indicating that variations in lipid composition significantly affect the size of the liposomes (Table S1). The effect of different lipid molar ratios and particle size could be defined from the following equation:

Particle size = 181.33A + 218.84B + 112.65C − 235.83D − 192.46AB + 98.46AC(4)

Increasing CH, DSPC, and DOTAP had a positive influence on liposome particle size (Figure 1A and Figure S1A). Higher CH can disrupt the regular packing of lipids, leading to larger vesicles due to increased rigidity and decreased curvature. In addition, DSPC is known to form relatively rigid bilayers. Therefore, increasing its concentration can enhance the rigidity of the liposome membrane, potentially leading to larger vesicle sizes due to the reduced flexibility required for forming smaller liposomes [64]. Higher DOTAP concentrations can disrupt the regular packing of phospholipids, leading to increased membrane fluidity and reduced bilayer rigidity. This can cause the formation of larger, more loosely packed liposomes because the membrane components are not as tightly constrained. In addition, higher DOTAP content can lead to a thicker hydration shell around the liposome, contributing to an increase in the overall size [65]. On the contrary, liposome particle size was inversely proportional to the C16-PEG2000 molar ratio. This result could be attributed to the steric hindrance associated with PEG that could decrease particle size [66]. Similar results were reported by Qin et al., during the preparation of different nucleic acid-loaded lipid nanoparticles [67]. The measured PDI of all formulae was less than 0.2 indicating the formation of the monodisperse homogenous system [33].

The zeta potential, which indicates the surface charge of the liposomes, ranged from 5.8 ± 0.4 mV to 8.1 ± 0.9 mV (Table S1). Equation (5) elucidates the impact of various lipids on the surface charge of liposomes, a critical parameter in their stability and biological interactions. CH, DSPC, and DOTAP each contribute positively to the zeta potential, enhancing the overall charge and allowing the encapsulation of siRNA [68]. In contrast, C16-PEG2000 significantly reduces the zeta potential, likely imparting stealth properties by lowering the surface charge (Figure 1B and Figure S1B) [69]. Notably, the interaction between DSPC and C16-PEG2000 demonstrates a synergistic effect, balancing the structural integrity and charge modulation. This interplay highlights the importance of lipid composition in tailoring liposome characteristics for specific therapeutic applications.

Zeta potential = 8.83A + 9.09B + 5.31C − 63.61D + 8.87BD (5)

Table S1 reveals that Sel EE% ranged from 46.29 ± 6.3% to 91.15 ± 4.3%. Analysis of Equation (6) and Figure 1C and Figure S1C indicates that all lipid components, particularly C16-PEG 2000, significantly enhance Sel EE%, likely due to their stabilizing effects on the liposomal structure and their ability to improve drug incorporation. However, the interaction between CH and DOTAP, as evidenced by the negative coefficient in the interaction term, suggests that high concentrations of these two lipids may negatively impact Sel EE%. The primary factor affecting liposome EE% is the stability of the lamellar structures and bilayer lipid configuration [70]. DSPC contributes to the formation of liposomes with a more rigid, spherical structure. CH within the liposomal membrane increases the spacing between phospholipid tails [64], promoting a more favorable lipid conformation. However, when CH concentration surpasses a certain threshold, excessive spacing between the phospholipid tails can occur, potentially leading to membrane disruption.

Sel EE% = 95.99A + 41.46B + 96.94C + 191.11D − 108.69AC (6)

Figure 1D and Figure S1D and Equation (7) illustrate the complex interplay between different lipid components in determining siRNA EE% within liposomes. CH, DSPC, and DOTAP each contribute positively to siRNA EE%, indicating their roles in enhancing encapsulation, likely through stabilizing the liposomal membrane and promoting siRNA incorporation. However, C16-PEG2000 has a substantial negative impact on siRNA EE%. Notably, Equation (6) highlights significant interaction effects, particularly the positive interactions between DSPC and C16-PEG2000 (BD), and between CH and C16-PEG2000 (AD), which dramatically enhance siRNA EE%. These interactions suggest that while certain lipid components might individually reduce EE%, their combined effects in specific ratios can significantly improve the overall siRNA encapsulation. This underscores the importance of optimizing lipid ratios to maximize the EE% of siRNA in liposomal formulations.

siRNA EE% = 77.54A + 58.11B + 73.57C − 422.13D + 30.93AB + 494.47AD + 27.92BC + 586.43BD + 584.78CD(7)

### 3.2. Optimal Lipid Compositions of Liposome for Sel and siRNA Delivery

The design space was determined by superimposing the effects of various factors on the contour plots of the investigated responses to achieve the smallest possible particle size while maximizing zeta potential, Sel EE%, and siRNA EE% (Figure S2A). The contour plots shown in Figure S2B–E illustrate the interactive effects of the formulation components on the predicted desirability of the final formulations. Based on the highest desirability score of 0.977, one formulation, referred to as Lip_Sel-siNEG_, was selected as a checkpoint to validate the statistical models. This formulation consists of CH, DSPC, DOTAP, and C16-PEG 2000 in a molar ratio of 40:15.15:40:4.85, respectively. The predicted and experimental values for particle size (Y1), zeta potential (Y2), Sel EE% (Y3), and siRNA EE% (Y4) are detailed in Table 1 and Table S2. The experimentally measured values for the Lip_Sel-siNEG_ formulation closely aligned with the predicted outcomes, with % error <7%. These results validate the model’s effectiveness in predicting the formulation’s physicochemical properties, indicating that the Lip_Sel-siNEG_ formulation is well-suited for delivering Sel and siRNA in therapeutic applications.

### 3.3. HSA Coated Liposomes Loaded Sel and siRNA

**Figure 2:**
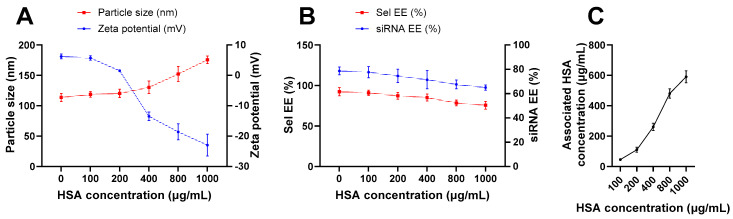
**The effect of lipid albumin coating on physicochemical characters of the optimized Lip_Sel-siNEG_.** Coating Sel-siRNA-loaded liposomes resulted in increased particle size and surface negativity (**A**). The EE% of both Sel and siRNA is inversely proportional to the concentration of HSA used in the coating (**B**). The amount of associated HSA in the optimized liposomes is quantified by Bicinchoninic Acid Protein Assay (BCA) (**C**). Data are represented as the mean ± SD (n = 3). The amount of associated HSA is directly proportional to HSA concentration.

Figure 2 shows the impact of HSA coating on the optimized Lip_Sel-siNEG_ physicochemical characters. Figure 2A shows a direct effect of HSA concentration and particle size. Initially, the particle size remains relatively stable up to around 400 µg/mL of HSA. Beyond this concentration, there is a noticeable increase in particle size, indicating that higher HSA concentrations result in larger particles [71]. An obvious decrease in zeta potential is noticed as the HSA concentration increases, indicating a more negative surface charge with higher HSA concentrations [72]. This trend suggests successful HSA adsorption on the nanoparticle surface (Figure 2A). Both Sel and siRNA EE% remain relatively stable with only a slight decrease as HSA concentration increases up to 400 µg/mL (Figure 2B). However, further increasing in HSA concentration of more than 400 µg/mL significantly diminish both Sel and siRNA EE% (*p* < 0.05). The amount of HSA associated with the liposome surface is positively affected when the HSA concentration increases (Figure 2C). There is a clear linear increase, indicating that higher initial concentrations of HSA result in greater association with the liposomes. This trend confirms that HSA is being successfully adsorbed onto the nanoparticle surface in a concentration-dependent manner. These findings highlight the importance of optimizing HSA concentration in liposome coating to achieve desired characteristics for drug delivery applications. Previous studies have shown that different nanocarriers with particle sizes up to 150 nm are able to traverse BBB directly via the nasal route [73]. Therefore, HSA in a concentration of 400 µg/mL was selected and the optimized formula would be referred to as C-Lip_Sel-siNEG_. Table 2 demonstrates the in vitro characterization of the fabricated C-Lip_Sel-siNEG_. Coating the optimized liposomes with human HSA adjusted the physicochemical properties significantly. The resultant HSA-coated liposomes, designated as C-LipSel-siNEG, exhibited an increased particle size of 136.5 ± 10.3 nm and a more negative zeta potential of −13.5 ± 1.4 mV. The EE% for Sel and siRNA slightly decreased to 85.12 ± 4.3% and 71.36 ± 7.5%, respectively, likely due to the dynamic changes induced by the protein coating on the liposomal membrane integrity and permeability [14].

### 3.4. In Vitro Serum Stability Study

**Figure 3:**
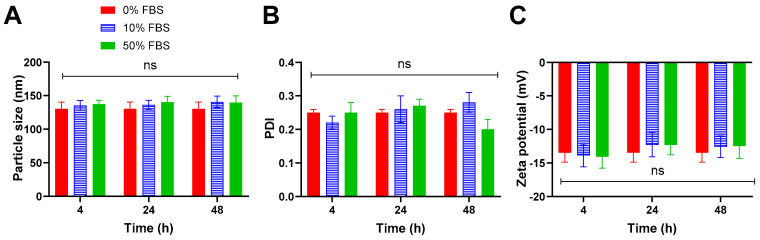
**Stability results of particle size, PDI, and zeta potential.** The selected C-Lip_Sel-siNEG_ was incubated with 0%, 10%, and 50% *v*/*v* FBS for 4, 24, and 48 h then particle size (**A**), PDI (**B**), and Zeta potential (**C**) were measured using DLS as described. Data points represent mean and SD (n = 3). Statistical analysis was carried out using one-way ANOVA followed by Tukey HSD test and *p <* 0.05 was considered significant, ns: non-significant. Serum protein had a non-significant effect on albumin-coated liposome particle size, PDI, or Zeta potential.

Variations in particle size, PDI, and zeta potential of C-Lip_Sel-siNEG_ were evaluated following incubation with 10% and 50% *v*/*v* FBS for 4, 24, and 48 h (Figure 3A–C). The serum has an insignificant effect on all tested parameters (*p* > 0.05). These results implied the insignificant effect of serum proteins on C-Lip_Sel-siNEG_ characters that could be ascribed to the electrostatic repulsion between the negative albumin coat charge and serum proteins [74].

### 3.5. In Vitro Sel Release Study

**Figure 4:**
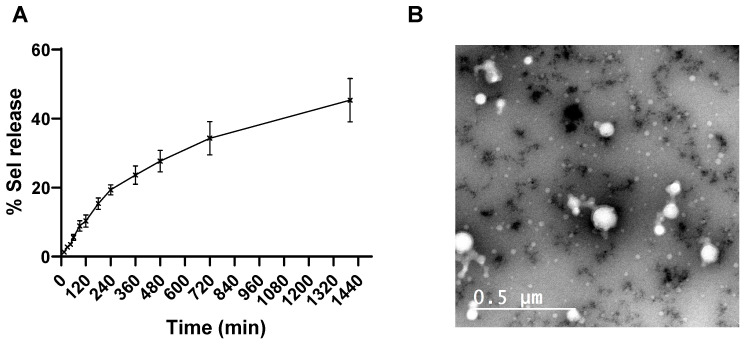
**In vitro characterization of the optimized C-Lip_Sel-siNEG._** In vitro release profile of Sel from the optimized C-Lip_Sel-siNEG_ in simulated nasal fluids at 35 °C (**A**). Drug release from liposomes is measured by dialyzing C-Lip_Sel-siNEG_ against simulated nasal fluids (pH 7.4). Drug concentration in the dialysate is assessed by HPLC. Datapoint represents mean and SD (n = 3). Morphological characterization of the optimized C-Lip_Sel-siNEG_ by Transmission Electron Micrography (**B**). C-Lip_Sel-siNEG_ appeared as spherical non-aggregate nanostructure.

The in vitro release profile of Sel from the optimized C-Lip_Sel-siNEG_ formulation was assessed at various time points over a period of 1400 min. The cumulative percentage release of Sel at each time point is presented in Figure 4A. The in vitro release data indicate a gradual and sustained release of Sel from the C-Lip_Sel-siNEG_ formulation over time. The release profile demonstrates an initial burst release within 120 min where approximately 10.32 ± 1.78% of Sel is released. This is followed by a slower, more controlled release phase, reaching 45.32% cumulative release at the end of 1400 min. The initial burst release can be attributed to the release of Sel that is loosely bound to the surface of the liposomes or present in the outer layers [75]. The subsequent sustained release phase suggests that the encapsulated Sel is being released in a controlled manner from the liposomal core. This biphasic release pattern is advantageous for maintaining therapeutic drug levels over an extended period, reducing the frequency of administration.

### 3.6. Transmission Electron Microscopy (TEM)

TEM micrograph of the optimized C-Lip_Sel-siNEG_ is illustrated in Figure 4B where spherical non-aggregated vesicles with a particle size range of 130–140 nm were detected and in agreement with the DLS results.

### 3.7. Cytotoxicity, Intracellular Uptake, and Silencing Efficiency of the Selected C-Lip_Sel-siSNCA2_ Formulation

**Figure 5:**
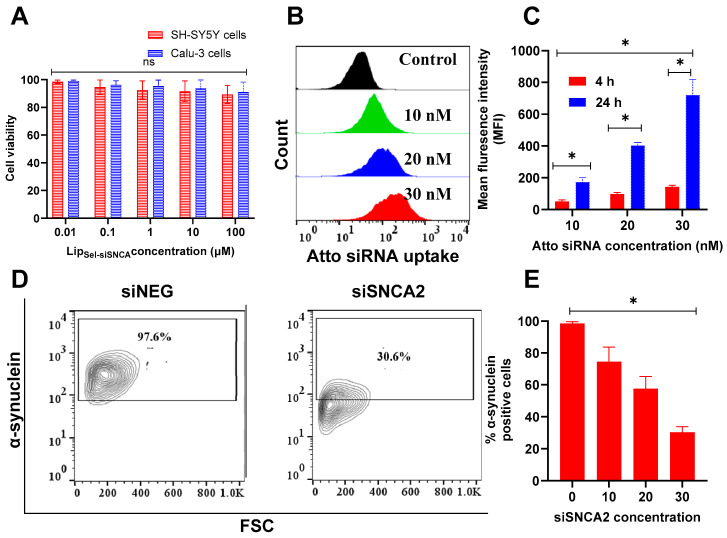
**Cytotoxicity, intracellular uptake, and silencing efficiency of C-Lip_Sel-siSNCA2_ in SH SY5Y cells.** SH-SY5Y cells were pretreated with rotenone (10 nM) for 5 days to induce α-synuclein overexpression. Consequently, cells were incubated with C-Lip_Sel-siSNCA2_ for 48 h at increasing Sel concentrations (0.01–100 μM). Cell viability was determined by MTT assay and data are presented as viable cells as a percentage of non-treated cells (n = 5) (**A**). The biocompatibility of the optimized C-Lip_Sel-siSNCA2_ was assessed also on Calu-3 cells. The optimized C-Lip_Sel-siSNCA2_ has an insignificant effect on the cell viability of both cells (ns: nonsignificant). Intracellular delivery of C-Lip_Sel-siAtto655_ at concentrations 10, 20, and 30 nM after 4 and 24 h was assessed using flow cytometry. Representative flow cytometry histograms obtained at the 4 h time point are shown in (**B**). Quantitative uptake of siRNA expressed as MFI is shown in (**C**). siRNA uptake was higher at increasing concentrations and incubation times (* *p* < 0.05). To evaluate gene silencing, SH-SY5Y cells pretreated with rotenone were incubated with C-Lip_Sel-siSNCA2_ at three different siSNCA_2_ concentrations (10, 20, and 30 nM) for 48 h. Representative flow cytometry contour plot for 30 nM siRNA at 48 h is shown in (**D**). Gates were drawn based on isotype controls. The knock-down efficiency of α-synuclein is presented as % positive cells (**E**). Data points represent mean and SD (n = 3). Statistical analysis was performed using One-way ANOVA followed by Tukey’s post-test * *p* < 0.05.

The investigation of the potential carrier toxicity is a prerequisite step for the clinical translation of any nanocarrier to evaluate the maximum tolerable dose using MTT assay [76]. An obvious high cell viability exceeding 90% was obtained up to a concentration equivalent to 100 µM for 48 h for all C-Lip_Sel-siSNCA2_ tested concentrations on both Calcu-3 and SH-SY5Y cells (Figure 5A). The Calu-3 cell line, derived from human airway epithelial cells, was chosen to model the nasal epithelial barrier, given the IN administration employed in this study. Calu-3 cells are widely recognized for their utility in evaluating drug permeability and cytotoxicity in nasal drug delivery applications. Furthermore, SH-SY5Y cells, derived from human neuroblastoma, serve as an established in vitro model for studying neurodegenerative diseases, including PD. Their inclusion allowed for the assessment of biocompatibility and safety of the liposomal system, ensuring its suitability for non-invasive delivery across the nasal mucosa. The optimized C-Lip_Sel-siNEG_ formula showed high cell viability at all tested drug concentrations after the incubation period (Figure 5A). The prepared system is formed from biocompatible lipids and HAS [77,78]. Several literatures discussed liposomes as a safe delivery system [79,80,81].

The ability of the optimized HSA-coated liposome to improve the internalization of siAtto655 in SH-SY5Y cells was assessed using flow cytometry across various concentrations (10–30 nM) and incubation times (4 and 24 h). The data expressed in Figure 5B,C revealed a pronounced dependency on both concentration and incubation time. At 4 h, the MFI value of 51.33 ± 7.97 at 10 nM was modest, indicating initial uptake was limited. However, at higher concentrations of 20 and 30 nM, there was a marked increase in MFI (96.66 ± 8.79 and 143 ± 7.87, respectively), suggesting more efficient siRNA internalization. After 24 h, all concentrations showed significant increases in MFI, with the highest value observed at 30 nM (720 ± 81.68), indicating prolonged exposure facilitated substantial siRNA uptake. This trend suggests that both higher siRNA concentrations and extended incubation times enhance cellular uptake, likely due to increased interactions with internalization mechanisms such as endocytic pathways [82]. These results agree with previous reports which state the nanocarriers’ uptake is usually time and concentration-dependent [75]. Generally, The optimized formula particle size as well as the presence of ligands such as HSA on the surface of cell delivery systems influences their internalization efficiency and method of internalization [83]. Efficient uptake of siRNA is crucial for its function in gene silencing, as the siRNA must enter the cytoplasm to interact with the RNA-induced silencing complex [84]. The observed increase in uptake with higher concentrations and longer incubation times suggests that optimizing these parameters can significantly enhance the delivery efficiency of siRNA. α-synuclein is implicated in the pathogenesis of PD and other synucleinopathies. Effective silencing of α-synuclein expression through siSNCA2 could offer a therapeutic strategy to mitigate disease progression [85]. The gene silencing efficiency of C-Lip_Sel-siSNCA2_ was evaluated by measuring the percentage of α-synuclein positive SH-SY5Y cells after treatment with different concentrations of siSNCA2 (10–30 nM). Incubation of SH-SY5Y cells with C-Lip_Sel-siNEG_ minimally affected α-synuclein expression (Figure 5D). On the contrary, a significant reduction in α-synuclein was observed at all concentrations (Figure 5D,E). Using the proposed system, α-synuclein positive cells were significantly reduced from 74.5 ± 9.2% to 30.3 ± 3.5% with increasing siSNCA2 concentration from 10 to 30 nM (*p* < 0.5). The obtained data illustrate a clear dose-dependent reduction in α-synuclein positive cells with increasing concentrations of siSNCA2.

### 3.8. Pharmacokinetics Sel Profile Following IN C-Lip_Sel-siSNCA2_

**Figure 6:**
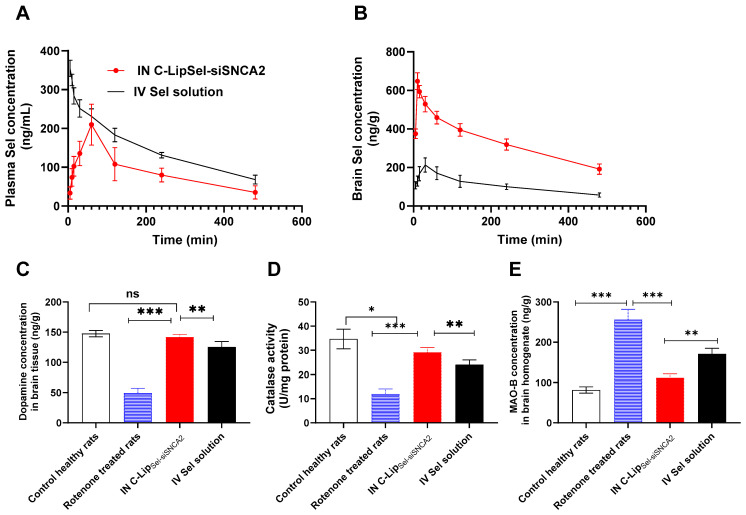
**Pharmacokinetics Sel profile and biochemical markers following administration of IN C-Lip_Sel-siSNCA2_ and intravenous Sel solution.** Sel concentrations in rats’ plasma (**A**) and brain (**B**) after administration of various formulations. Animals received Sel at a dose of 1 mg/kg either via IV injection through the tail vein or IN instillation of 10 µL of C-Lip_Sel-siSNCA2_ containing 0.75 mg/kg siSNCA2 in each nostril. At each time point, 6 animals were sacrificed from each group and the concentration of Sel in plasma and brain was quantified using LCMS/MS. A significantly higher brain Sel concentration was observed at all time points following IN administration of C-Lip_Sel-siSNCA2_ compared to IV solution. Datapoints represent the mean ± SE (n = 6). PD was simulated in rats by IP injection of rotenone (3 mg/kg/day) for 11 days. On days 12 and 20 post-starting rotenone injection, rats received either IN C-Lip_Sel-siSNCA2_ or IV Sel solution. On day 30 of the experiment, rats were sacrificed and brain tissues were collected. Brain tissues were homogenized and the concentration of dopamine in the brain homogenate was assessed by HPLC-equipped with a fluorescence detector set at excitation/emission wavelengths of 280/315 nm (**C**). Catalase activity in the brain tissue homogenate was measured by a colorimetric method at 570 nm (**D**). MAO-B level was quantified in brain tissue using ELISA (**E**). Data points represent mean and SE (n = 6). Statistical analysis was performed using Two-way ANOVA followed by Tukey’s post-test, ns: non-significant, * *p* < 0.05, ** *p* < 0.01, *** *p* < 0.001. IN C-Lip_Sel-siSNCA2_ administration successfully restored dopamine levels, while catalase activity significantly reduced MAO-B levels in Parkinson’s model rats’ brains.

To further validate the obtained in vitro findings, it is essential to conduct in vivo studies to assess the pharmacokinetics and pharmacodynamics of the IN C-Lip_Sel-siSNCA2_ formulation. These studies will help to confirm the bioavailability and therapeutic efficacy of the formulation in a biological system. The pharmacokinetic parameters following IN C-Lip_Sel-siSNCA2_ and IV Sel solution administration were evaluated in both plasma and brain tissues (Figure 6A,B and Table 3). In plasma, the C_max_ of IN C-Lip_Sel-siSNCA2_ was 0.21 ± 0.03 µg/mL with T_max_ 60 min. The calculated AUC_0–480min_ for IN C-Lip_Sel-siSNCA2_ and IV Sel solutions were 0.71 ± 0.06 and 1.16 ± 0.09 µg/mL·h, respectively. The respective AUC_0–∞_ for IN C-Lip_Sel-siSNCA2_ and IV Sel solution was 0.9 ± 0.14 and 1.54 ± 0.21 µg/mL·h. The calculated MRT for IN C-Lip_Sel-siSNCA2_ was 4 ± 0.99 h with K_el_ of 0.18 ± 0.01 h^−1^ compared to 4.22 ± 0.84 h and 0.17 ± 0.02 h^−1^ for IV Sel solution. The absolute bioavailability (F%) of IN C-Lip_Sel-siSNCA2_ was calculated to be 61.2%.

In the brain, IN C-Lip_Sel-siSNCA2_ showed a C_max_ of 0.65 ± 0.09 µg/mL, which is significantly higher than the C_max_ of 0.21 ± 0.03 µg/mL for the IV Sel solution (*p* < 0.05). The T_max_ for IN C-Lip_Sel-siSNCA2_ was 10 min, compared to 30 min for the IV Sel solution, indicating a faster peak concentration achievement for the IN route and suggesting a rapid onset of action. The AUC_0–480min_ for IN C-Lip_Sel-siSNCA2_ was 2.64 ± 0.11 µg/mL·h, whereas it was 0.85 ± 0.06 µg/mL·h for the IV Sel solution. The AUC_0–∞_ for IN C-Lip_Sel-siSNCA2_ was 4.04 ± 0.68 µg/mL·h, significantly higher than 1.22 ± 0.15 µg/mL·h for the IV Sel solution, demonstrating greater overall brain deposition with IN administration. The significantly higher C_max_ and AUC values for IN C-Lip_Sel-siSNCA2_ compared to the IV Sel solution indicate better brain penetration and prolonged exposure which is crucial for therapeutic interventions in neurodegenerative conditions like PD. The use of liposomal formulation and the nano vesicle size enables the deeper transport of drug particles into the layers of olfactory epithelial cells [39,51] facilitating their easy translocation between different cerebral compartments [40]. In addition, albumin coating on the liposomes likely contributes to their stability and prolongs their residence time in the nasal cavity, facilitating sustained release and absorption of the drug. A slower elimination of Sel from the brain was noticed following IN C-Lip_Sel-siSNCA2_ compared to IV Sel solution as indicated with MRT values of 4.88 ± 0.62 h and 4.56 ± 0.75 h, respectively. Moreover, the K_el_ for IN C-Lip_Sel_-siSNCA2 was 0.13 ± 0.013 h^−1^, which is lower than 0.16 ± 0.014 h^−1^ for the IV Sel solution suggesting sustained release and prolonged retention of the drug, enhancing its therapeutic potential. The DTE for IN C-Lip_Sel-siSNCA2_ was calculated to be 507.43%, indicating highly efficient targeting of the brain. In addition, the calculated DTP was 80.29%, showing a substantial portion of the drug reaching the brain directly via the IN route as previously suggested by various studies [51,56,57]. This efficient transport mechanism enhances drug absorption and accumulation directly in the brain [51]. The exceptionally high DTE and DTP values underscore the effectiveness of IN administration in targeting the brain, minimizing systemic exposure, and potentially reducing side effects. The IN route provides a direct pathway to the brain via the olfactory and trigeminal nerve pathways, circumventing the BBB. This direct nose-to-brain access not only enhances the efficacy of drug delivery but also reduces the systemic exposure and potential side effects associated with the drug.

### 3.9. Biomarker Analyses in Parkinson’s Model Rats

Dopamine concentration and catalase activity in brain tissues, following the administration of IN C-Lip_Sel-siSNCA2_ and IV Sel solution in a PD rat model, were quantified (Figure 6C,D). The dopamine levels in brain tissue were significantly reduced in the rotenone-treated rats compared to control healthy rats, indicating successful induction of PD [86]. The dopamine concentration in the control healthy rats was 147.5 ± 2.14 ng/g, while in the rotenone-treated rats, it dropped significantly to 49.66 ± 3.23 ng/g (*p* < 0.001). However, the administration of IN C-Lip_Sel-siSNCA2_ significantly restored dopamine levels to 142 ± 1.83 ng/g, which is close to the levels in control healthy rats. The IV Sel solution also increased dopamine levels to 125.33 ± 3.71 ng/g, but this increase was not as pronounced as the IN treatment and was statistically non-significant compared to rotenone-treated rats (Figure 6C). The significant restoration of dopamine levels in the brain tissue of IN C-Lip_Sel-siSNCA2_ treated rats highlights the potential of this IN delivery system for neuroprotection and symptomatic relief in PD. The IN route facilitates direct delivery to the brain, bypassing the BBB, and achieving higher and more effective concentrations of the therapeutic agent. The near-normalization of dopamine levels indicates that IN C-Lip_Sel-siSNCA2_ can effectively counteract the dopamine depletion caused by rotenone, which is a hallmark of PD pathology. In contrast, the IV Sel solution, while also improving dopamine levels, was less effective than the IN route. This suggests that systemic administration may not be as efficient in delivering sufficient concentrations of the drug to the brain, likely due to the limitations imposed by the BBB and systemic metabolism. Catalase activity, an important antioxidant enzyme, was also assessed. The activity was significantly reduced in the rotenone-treated rats (12 ± 0.85 U/mg protein) compared to control healthy rats (34.66 ± 1.67 U/mg protein), reflecting oxidative stress associated with PD. IN C-Lip_Sel-siSNCA2_ treatment significantly increased catalase activity to 29.16 ± 0.79 U/mg protein, indicating a strong antioxidative effect compared to rotenone treated group (*p* < 0.001). IV Sel solution also improved catalase activity to 24 ± 0.82 U/mg protein, which was significantly higher than the rotenone-treated group (*p* < 0.05), but lower than the IN C-Lip_Sel-siSNCA2_ group (*p* < 0.01) (Figure 6D). The significant increase in catalase activity following IN C-Lip_Sel-siSNCA2_ treatment underscores its antioxidative properties. Catalase is crucial in mitigating oxidative stress, which is a major contributing factor to neuronal degeneration in PD. The ability of IN C-Lip_Sel-siSNCA2_ to restore catalase activity to near-normal levels indicates a robust antioxidative defense mechanism, protecting neurons from oxidative damage and promoting overall brain health [87]. The IV Sel solution also showed beneficial effects on catalase activity, but again, the response was less pronounced compared to the IN treatment. This further supports the superiority of the IN route for targeting brain tissues effectively. When exposed to rotenone, the protein level of MAO-B in the brain tissues increased significantly (256.33 ± 25.6 ng/g) in comparison to the negative control (81.66 ± 7.71 ng/g) (*p* < 0.001) (Figure 6E). As the brain ages, MAO-B, a marker of increased dopamine metabolism in the synaptic cleft, rises, which may be the cause of an increased prevalence of PD in the elderly. When the brain’s MAO-B levels are high, dopamine in the synaptic cleft is rapidly converted to dihydroxyphenylacetic acid. The latter is known to reduce dopamine receptor activation by causing Lewy body development and a-synuclein aggregation, which are characteristic molecular markers of PD [60,88]. As shown in Figure 6E, the group treated with C-Lip_Sel-siSNCA2_ had a significantly lower level of MAO-B (112.33 ± 9.62 ng/g) than the group treated with IV Sel solution (170.83 ± 13.93 ng/g) (*p* < 0.01). Following IN therapy, there is a notable and substantial decrease in the amount of MAO-B (the enzyme itself), which guarantees that α-synuclein does not aggregate. The brain targets and dual action of C-Lip_Sel-siSNCA2_; the Sel MAO-B inhibitor effect; and siRNA controls α-syngene overexpression, when compared to IV injection. The biochemical analysis of dopamine concentration, catalase activity, and MAO-B in brain tissues provides compelling evidence for the efficacy of IN C-Lip_Sel-siSNCA2_ in mitigating PD symptoms in the rat model.

### 3.10. Pharmacodynamic Assessment of C-Lip_Sel-siSNCA2_ in Parkinson’s Model Rats

**Figure 7:**
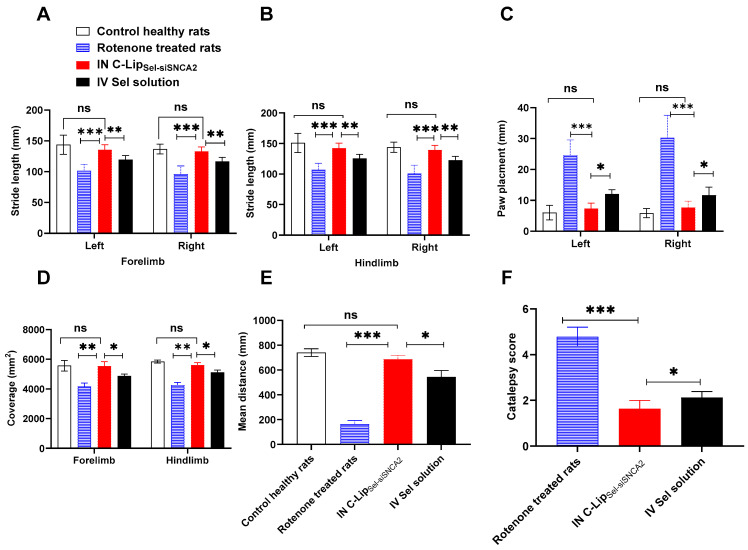
**Pharmacodynamic assessment of C-Lip_Sel-siSNCA2_ Parkinson’s model rats.** PD was simulated in rats by IP injection of rotenone (3 mg/kg/day) for 11 days. On day 12 and 20 post starting rotenone injection, rats received either IN C-Lip_Sel-siSNCA2_ or IV Sel solution. On day 30 of the experiment, stride length in left and right Forelimb and Hind limbs (**A**,**B**), Paw placement (**C**), and coverage (**D**) were measured and compared to control healthy rats and rotenone-treated rats. The open field test was carried out over 60 min and the distance crossed by rats was recorded (**E**). Catalepsy score was measured using bar method by measuring the time required by the rat to remove its forepaws from the rod (**F**). The rats were scored using a 5-point scale; 0 (0–5 s), 1 (6–15 s), 2 (16–25 s), 3 (26–35 s), 4 (36–60 s), and 5 (>60 s). Data points represent mean and SE (n = 6). Statistical analysis was performed using Two-way ANOVA followed by Tukey’s post-test, ns: non-significant, * *p* < 0.05, ** *p* < 0.01, *** *p* < 0.001. IN C-Lip_Sel-siSNCA2_ administration successfully restored locomotor activity in Parkinson’s model rats.

PD was simulated in rats via IP injection of rotenone (3 mg/kg/day) for 11 days. Treatments were administered on days 12 and 20 post-rotenone injections, with rats receiving either IN C-Lip_Sel-siSNCA2_ or IV Sel solution. The locomotor activities were assessed on day 30, including stride length in the forelimb and hindlimb, paw placement, coverage area, distance traveled in the open field test, and catalepsy score (Figure 7).

All pharmacodynamic parameters were compared to healthy rats (on day 1 before any treatment) as well as the untreated PD simulated rat model (on day 11 after daily rotenone administration) [53]. PD is associated with gait difficulty which is initially signed by motor dysfunction as walking difficulties. As expected, rotenone-induced neuronal injury resulted in a significant decrease in stride lengths in both the forelimb and hindlimb (*p* < 0.001) (Figure 7A,B).

On the contrary, animals received IN C-Lip_Sel-siSNCA2_ showed a significantly higher stride length than those received either IV Sel solution or rotenone solution (*p* < 0.05) with respective values of 135.66 ± 3.22, 119.5 ± 2.66, 102 ± 4.2 mm for left forelimb. Similarly, the measured stride length in the right forelimb, and right and left hindlimb in rats treated with IN C-Lip_Sel-siSNCA2_ was significantly higher than that measured in animals received IV Sel solution or rotenone solution (*p* < 0.05). Additionally, IN C-Lip_Sel-siSNCA2_ succeeded in restoring comparable stride length values to those observed in normal healthy rats (*p* > 0.05). Figure 7C illustrates the measured paw placement of animals received either IN C-Lip_Sel-siSNCA2_, IV Sel solution, or rotenone solution in comparison to healthy rats. The significant restoration of stride length in both forelimbs and hindlimbs indicates enhanced motor coordination and muscle function. This improvement in stride length suggests that IN C-Lip_Sel-siSNCA2_ treatment can effectively counteract the motor deficits induced by rotenone.

Generally, relative paw placement was calculated to determine how the motor coordination and spatial awareness of the rats. In the rotenone-treated group, paw placement of both left and right sides (24.66 ± 2.02 mm and 30.33 ± 2.95 mm, respectively) was significantly impaired compared to the healthy control group (6 ± 0.96 mm and 5.83 ± 0.6 mm) (*p* < 0.001). This finding suggests that the same paw was placed repeatedly and that the distance between the fore and rear legs decreased, indicating gait anomalies like those seen in humans. This change represents the complete extent of the nigrostriatal lesion [89]. However, rats treated with IN C-Lip_Sel-siSNCA2_ exhibited significant improvements in paw placement accuracy for both left and right sides compared to those receiving IV solution with respective values of 7.33 ± 0.71 mm, 7.66 ± 0.88 mm, 12 ± 0.57 mm and 11.66 ± 1.05 mm (*p* < 0.05) (Figure 7C).

Under normal circumstances, healthy rats showed symmetric paw placement with a high coverage area of 5566.66 ± 145.29 mm^2^ and 5841.66 ± 41.66 mm^2^ for forelimb and hindlimb, respectively (Figure 7D). The total coverage area, which measures the area covered by the rats during the test period, was significantly reduced in the rotenone-treated group compared to the healthy control group. This reduction indicates impaired locomotor activity (*p* < 0.01). In contrast, rats treated with IN C-Lip_Sel-siSNCA2_ showed a coverage area of 5550 ± 117.61 mm^2^ and 5625 ± 61.57 mm^2^ for the forelimb and hindlimb, respectively. This significant increase in coverage area, compared to the rotenone group, suggests enhanced locomotor activity and improved motor function (Figure 7D). Interestingly, an insignificant difference in coverage area was noticed between rats treated with IN C-Lip_Sel-siSNCA2_ compared to healthy rats, but it was substantially greater than that measured following IV Sel-solution. Improved paw placement accuracy and increased coverage area further support the efficacy of IN C-Lip_Sel-siSNCA2_ in enhancing motor coordination and spatial awareness. The significant improvements in these parameters indicate that the treatment enhances fine motor skills and overall locomotor function.

The open field test measures the distance crossed by the rats over a 60-minute period, providing an indicator of exploratory behavior and overall motor function. Figure 7E shows a significant reduction in distance traveled by rats in the rotenone-treated group compared to healthy control rats (*p* < 0.001). The decrease in locomotor activity in rotenone-induced rats indicated functional changes in dopaminergic motor neurons [90]. Rats treated with IN C-Lip_Sel-siSNCA2_ showed a significant 4- and 1.2-fold increase in the distance traveled compared to the rotenone-treated group and IV Sel solution, respectively (Figure 7E). This increased movement distance indicates enhanced exploratory behavior and motor function in the IN C-Lip_Sel-siSNCA2_ treated rats (Figure 7E). Additionally, the results from the open field test measuring the number of center crossings in a 60-minute observation period reveal significant differences across experimental groups, reflecting anxiety-like behavior and exploratory tendencies. Control healthy rats demonstrated the highest mean number of center crossings (17.83 ± 1.57), indicating normal exploratory behavior and low anxiety levels. In contrast, rotenone-treated rats, modeling PD, exhibited a markedly reduced mean number of center crossings (3.00 ± 1.91) (*p* < 0.001), consistent with impaired motor function and heightened anxiety. Treatment with the obtained IN C-Lip_Sel-siSNCA2_ significantly improved exploratory behavior compared to rotenone-treated rats, with a mean of 12.33 ± 1.97 (*p* < 0.01), suggesting reduced anxiety and partial restoration of motor function. Similarly, the IV Sel solution group showed moderate improvement, recording 8.33 ± 1.49 crossings, although the effects were less pronounced than those observed with the IN formulation. These findings support the hypothesis that the IN C-Lip_Sel-siSNCA2_ formulation offers superior therapeutic outcomes, particularly in reducing anxiety-like behavior and improving motor function, compared to intravenous delivery [91].

In order to evaluate the rigidity and motor control of the rats, the catalepsy score was assessed. No signs of catalepsy scores could be noticed in normal healthy rats (Figure 7F). On the contrary, a catalepsy score of 4.78 ± 0.17, 1.63 ± 0.15, and 2.11 ± 0.11 for rats received rotenone injection, IN C-Lip_Sel-siSNCA2_ and IV Sel solution, respectively. The significantly lower catalepsy score in rats received IN C-Lip_Sel-siSNCA2_ compared to the other two groups indicated quicker removal of forepaws from the rod (*p* < 0.05), hence better motor control and reduced PD-related symptoms (Figure 7F). This suggests the functional preservation of dopaminergic neurons, potentially due to the protective impact on the activity of α/K ATPase [92]. Collectively, these findings suggest that IN C-Lip_Sel-siSNCA2_ is effective in ameliorating the motor deficits induced by rotenone in the PD rat model over IV Sel solution. Although rapid absorption occurs after IV administration, the brain targets and dual action of C-Lip_Sel-siSNCA2_ showed a superior therapeutic effect in terms of improved motor function and reduced PD symptoms, warranting further investigation and potential clinical application.

## 4. Conclusions

This study successfully demonstrates the development of a novel IN delivery system utilizing albumin-coated liposomes (C-Lip_Sel-siSNCA2_) for the co-delivery of Sel and α-synuclein-targeting siRNA, providing a promising therapeutic approach for PD. The optimized liposomal formulation, characterized by a particle size of 136.5 ± 10.3 nm and a negative zeta potential of −13.5 ± 1.4 mV, achieved high EE% for both Sel and siRNA. The fabricated stable C-Lip_Sel-siSNCA2_ improved cellular uptake and gene silencing of α-synuclein-targeting siRNA. It also significantly improved brain-targeting efficiency, as demonstrated by superior pharmacokinetic and pharmacodynamic profiles in a rotenone-induced PD rat model. The IN administration of C-Lip_Sel-siSNCA2_ led to a marked improvement in motor and non-motor function, and a substantial restoration of dopamine levels and catalase activity as well as reducing MAO-B levels, indicating its potential for mitigating PD symptoms. These findings underscore the therapeutic potential of the C-Lip_Sel-siSNCA2_ formulation as a non-invasive and effective treatment strategy for PD. This innovative approach could pave the way for further investigations into multifunctional nanocarriers capable of delivering combination therapies directly to the brain, offering hope for more effective and targeted treatment strategies in neurodegenerative diseases. Future studies will focus on comprehensive biological and toxicological evaluations to further validate the clinical applicability of this fabricated system.

## Figures and Tables

**Table 1:** Factors and responses in D-optimal design for Sel-siRNA loaded liposomes.

Factors	Levels	
	Low	High
A: CH ^a^	40	50
B: DSPC ^b^	10	20
C: DOTAP ^c^	25	40
D: C16-PEG2000 ^d^	2.5	5
Responses	Constrains	
Y1: Particle size (nm)	Minimize	
Y2: Zeta potential (mV)	Maximize	
Y3: Sel EE (%)	Maximize	
Y4: siRNA EE (%)	Maximize	

^a^ CH is cholesterol. ^b^ DSPC is 1,2-distearoyl-sn-glycero-3-phosphocholine. ^c^ DOTAP is Dioleoyl-3-trimethylammonium propane. ^d^ C16-PEG 2000 is N-palmitoyl-sphingosine-1-[succinyl (methoxypolyethylene glycol) 2000.

**Table 2:** Physicochemical characterization of the optimized liposomes and HSA-coated liposomes loaded Sel and siRNA.

Formula	Lipid Molar Composition (%) ^a^				HSA conc. (µg/mL) ^b^	Particle Size (nm) ^c,g^	Zeta Potential (mV) ^d,g^	Sel EE (%) ^e,g^	siRNA EE (%) ^f,g^
	CH	DSPC	DOTAP	C16-PEG2000					
Lip_Sel-siNEG_	40	15.15	40	4.85	-----	113.5 ± 6.8	6.2 ± 0.8	92.35 ± 5	78.66 ± 3.2
C-Lip_Sel-siNEG_					400	136.5 ± 10.3	−13.5 ± 1.4	85.12 ± 4.3	71.36 ± 7.5

^a^ total lipid is 0.22 µmole. ^b^ HSA is dissolved in PBS (pH 7.4) and added dropwise to the optimized liposomes at 37 °C for 1 h. ^c^ measured by dynamic light scattering technique after dilution in deionized water (1:10 *v*/*v*). ^d^ measured by electrophoresis technique after dilution in deionized water (1:10 *v*/*v*). ^e^ calculated directly as percentage of Sel added, determined by HPLC. ^f^ calculated directly as percentage of siRNA added, determined by RiboGreen assay. ^g^ expressed as mean ± SD (n = 3).

**Table 3:** Pharmacokinetic parameters of IN C-Lip_Sel-siSNCA2_ and intravenous Sel solution.

Parameter	Plasma		Brain	
	IN C-Lip_Sel-siSNCA2_	IV Sel Solution	IN C-Lip_Sel-siSNCA2_	IV Sel Solution
C_max_ (µg/mL)	0.21 ± 0.03	--------	0.65 ± 0.09	0.21 ± 0.03
T_max_ (min)	60	--------	10	30
AUC_0–480min_ (µg/mL·h)	0.71 ± 0.06	1.16 ± 0.09	2.64 ± 0.11	0.85 ± 0.06
AUC_0–∞_ (µg/mL·h)	0.9 ± 0.14	1.54 ± 0.21	4.04 ± 0.68	1.22 ± 0.15
MRT (h)	4 ± 0.99	4.22 ± 0.84	4.88 ± 0.62	4.56 ± 0.75
K_el_ (h^−1^)	0.18 ± 0.01	0.17 ± 0.02	0.13 ± 0.013	0.16 ± 0.014
Absolute bioavailability (F%)	61.2	100	--------	--------
DTE (%)	--------	--------	507.43	--------
DTP (%)	--------	--------	80.29	--------

## Data Availability

Data will be made available on request.

## Supplementary Materials

The following supporting information can be downloaded at: https://www.mdpi.com/article/10.3390/pharmaceutics17020243/s1, Table S1. Physicochemical characterization of liposomes loaded selegiline and siRNA in the designed formulations; Figure S1. The effect of lipid composition on particle size, zeta potential, Sel and siRNA entrapment efficiency. Figure S2. Optimal lipid compositions of liposome for selegiline and siRNA delivery; Table S2. The experimental and predicted physicochemical characterization of the optimized liposomes (LipSel-siSNCA2).
