# Supplementary material for: Tailored Intranasal Albumin Caged Selegiline-α Synuclein siRNA Liposome with Improved Efficiency in Parkinson’s Model
Source: Pharmaceutics. 2025 Feb 12;17(2):243. doi: 10.3390/pharmaceutics17020243 (PMC11859980; doi:10.3390/pharmaceutics17020243)
Supplement: Supplementary file 1 [file pharmaceutics-17-00243-s001.zip › pharmaceutics-3425544-supplementary.pdf]

Tailored intranasal albumin caged selegiline- α synuclein siRNA liposome with improved efficiency in Parkinson's model

Ahmed A. Katamesh [1,*,†], Hend Mohamed Abdel-Bar [2,*,†], Mohammed Khaled Bin Break [3,4], Shimaa M. Hassoun [5], Gehad Mohammed Subaiea [5], Amr Radwan [6,7] and Hadel A. Abo El-Enin [8,+]

[1] Department of Pharmaceutics, College of Pharmacy, University of Ha'il, Ha'il 81442, Saudi Arabia
[2] Department of Pharmaceutics, Faculty of Pharmacy, University of Sadat City, P.O. Box 32897, Menoufia, Egypt
[3] Department of Pharmaceutical Chemistry, College of Pharmacy, University of Ha'il, Ha'il 81442, Saudi Arabia; m.binbreak@uoh.edu.sa
[4] Medical and Diagnostic Research Centre, University of Ha'il, Ha'il 55473, Saudi Arabia
[5] Department of Pharmacology, College of Pharmacy, University of Ha'il, Ha'il 81442, Saudi Arabia; s.hassoun@uoh.edu.sa(S.M.), g.subaiea@uoh.edu.sa (G.S.).
[6] Research Department, Academy of Scientific Research and Technology, Cairo, 11694, Egypt; radwan.amro@gmail.com
[7] Egyptian Center for Innovation and Technology Development, Cairo, 11512, Egypt
[8] Department of Pharmaceutics, Egyptian Drug Authority, Giza 12511, Egypt; hadelaboenin@outlook.com (H.A.)
* Correspondence: hend.abdelbar@fop.usc.edu.eg (H.M.A.-B.); a.katamsh@uoh.edu.sa (A.A.K.).
† These authors contributed equally to this work.

Table S1. Physicochemical characterization of liposomes loaded Sel and siRNA in the designed formulations.

Run	Lipid molar composition (%) [a]				Particle size (nm) [b, f]	Zeta potential (mV) [c, f]	Sel EE (%) [d, f]	siRNA EE (%) [e, f]
	A: CH	B: DSPC	C: DOTAP	D: C16-PEG2000				
1	50	20	25	5	116.6±5.9	7.6±0.7	84.45±3.1	72.84±3.6
2	40	15	40	5	106.5±4.3	5.8±0.4	91.15±4.3	77.26±3.6
3	50	20	27.5	2.5	157.2±3.9	8.1 ±0.9	50.19±5.3	75.58±4.2
4	50	10	37.5	2.5	167.3±5.6	7.8±0.9	70.95±3.6	73.78±6.2
5	40	15	40	5	110.5±6.9	5.8±0.4	88.46±4.5	76.84±4.5
6	43.33	19.17	35	2.5	150.1±7.5	7.1±0.6	56.03±3.2	74.86±3.3
7	50	20	25	5	118.6±9.1	7.7±0.4	84.21±5.4	73.05±8.5
8	40	20	37.5	2.5	143.9±10.3	6.3±0.5	64.16±4.7	73.45±5.7
9	46.67	20	30.83	2.5	152.1±11.9	7.4±0.3	46.29±6.3	74.58±3.6
10	43.75	13.75	40	2.5	143.9±8.6	6.7±0.8	69.31±3.6	74.77±4.9
11	43.33	20	31.67	5	132.7±3.4	7.4±0.3	79.88±8.6	74.01±5.6
12	46.25	10	40	3.75	133.8±7.5	6.7±0.7	79.48±4.5	75.67±7.5
13	45	15	35	5	119.6±3.6	6.4±0.9	87.58±7.9	75.05±6.3
14	50	15	31.25	3.75	139.9±7.9	7.7±0.4	74.58±8.3	76.65±4.6
15	49.17	13.33	35	2.5	156.1±4.5	7.4±0.5	59.45±5.6	75.97±4.6
16	50	10	35	5	130.8±9.2	6.8±0.9	86.11±10.2	72.52±7.6
17	46.25	10	40	3.75	138.9±4.6	6.9±0.7	81.52±3.6	75.23±3.6
18	50	15	31.25	3.75	141.3±8.9	7.6±0.3	66.76±7.6	77.08±8.5
19	50	10	35	5	129.4±5.8	6.6±0.4	87.81±8.1	72.41±4.5
20	46.67	16.67	31.66	5	121.4±3.6	7.2±0.6	77.07±5.9	74.55±5.6

[a] total lipid is 0.22 μmole

[b] measured by dynamic light scattering technique after dilution in deionized water (1: 10 v/v)

[c] measured by electrophoresis technique after dilution in deionized water (1: 10 v/v)

[d] calculated directly as percentage of Sel added, determined by HPLC

[e] calculated directly as percentage of siRNA added, determined by RiboGreen assay

[f] expressed as mean ± SD (n=3)

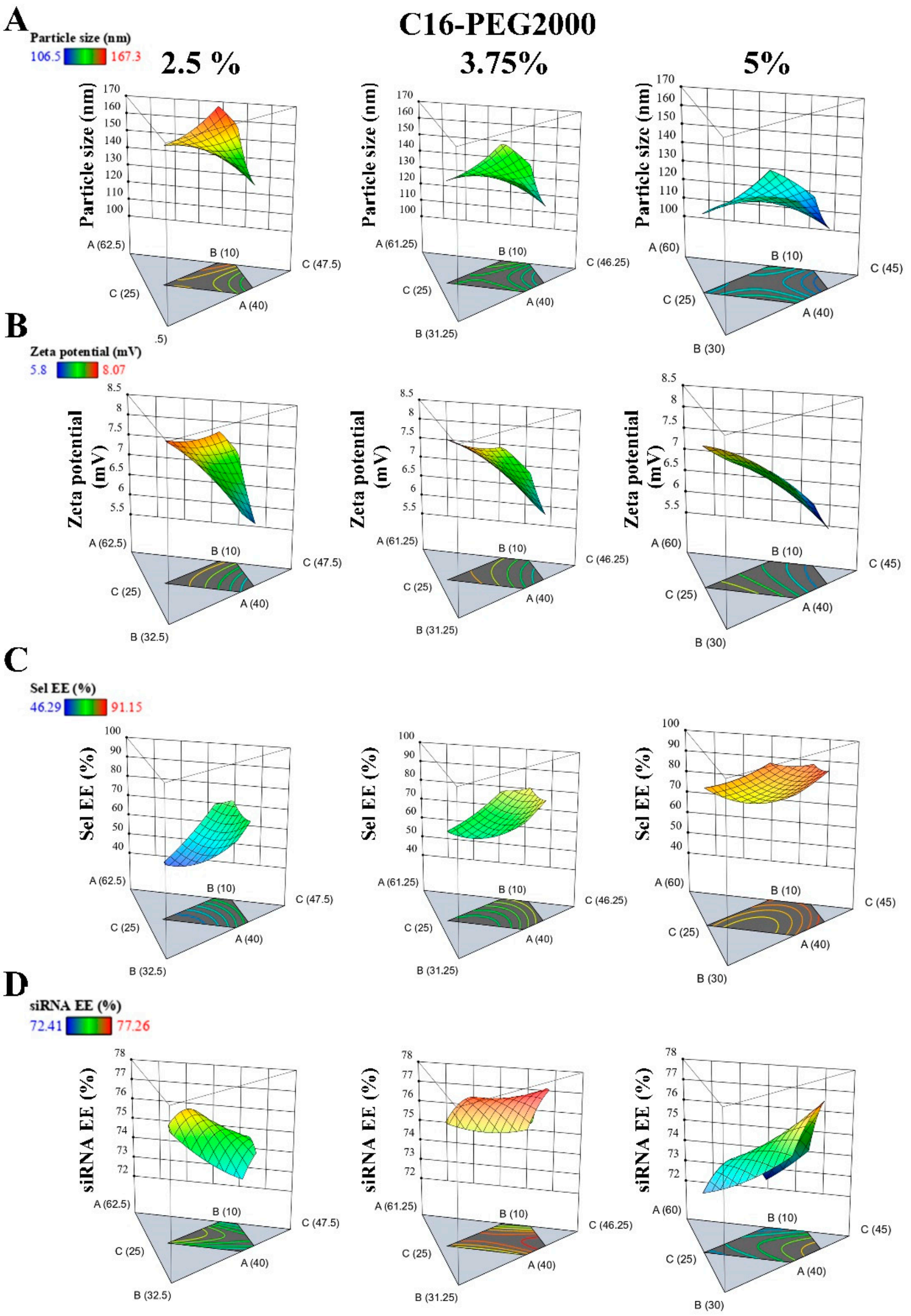

Figure S1. The effect of lipid composition on particle size, zeta potential, Sel and siRNA entrapment efficiency. Data obtained from physicochemical characterization of the prepared liposomes were used to generate a predictive DoE model. Lipid interactions defined between CH (A), DSPC (B) and DOTAP (C) when C16-PEG2000 (D) was used at 2.5% (left), 3.75% (middle), 5% (right) on particle size (Y1) (A), zeta potential (Y2) (B), Sel EE% (Y3) (C) and siRNA EE% (Y4) (D) are presented as 3D plots.

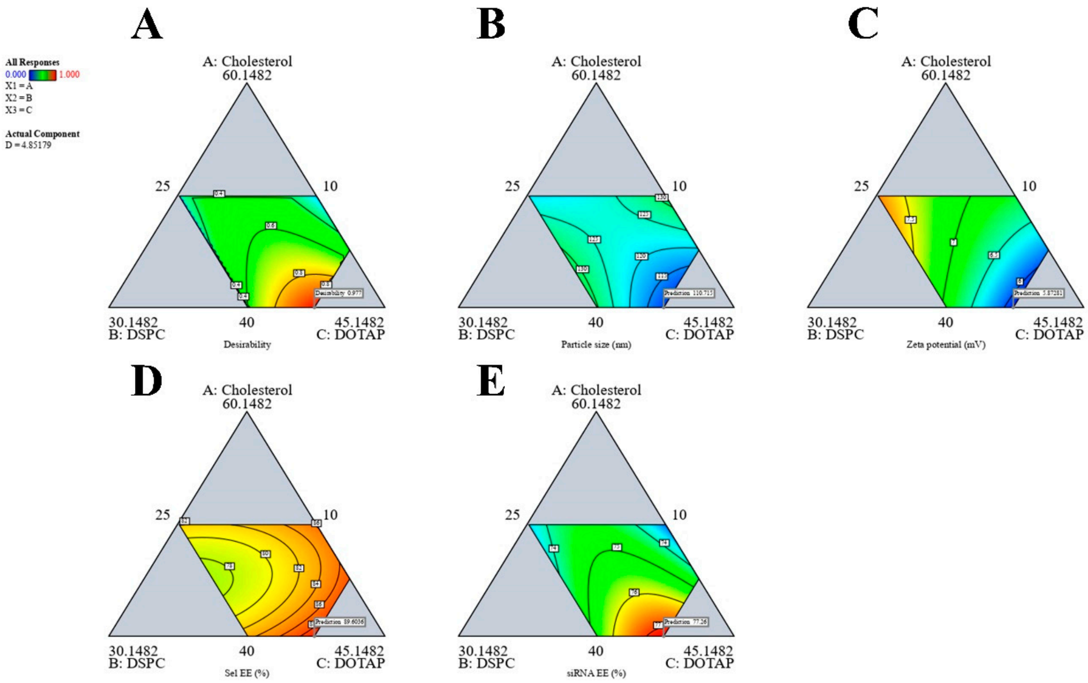

Figure S2. Optimal lipid compositions of liposome for Sel and siRNA delivery. Lipid interactions defined between CH (A), DSPC (B) and DOTAP (C) when C16-PEG2000 (D) was used at 4.85% on desirability (A), particle size (Y1) (B), zeta potential (Y2) (C), Sel EE% (Y3) (D) and siRNA EE% (Y4) (E) are presented as contour plots.

Table S2. The experimental and predicted physicochemical characterization of the optimized liposomes (Lip$_{Sel\text{-}siSNCA2}$) [a]

Parameter	Experimental	Predicted	% Predicted error
Particle size (nm) [b, f]	113.5± 6.8	110.7	2.46
Zeta potential (mV) [c, f]	6.2±0.8	5.8	6.45
Sel EE (%) [d, f]	92.35±5	89.6	2.97
siRNA EE (%) [e, f]	78.66±3.2	77.26	1.78

[a] total lipid is 0.22 µmole and the optimized formula Lip$_{Sel\text{-}siSNCA2}$ is composed of CH: DSPC: DOTAP: C16-PEG 2000 in a molar ratio of 40: 15.15: 40: 4.85 respectively

[b] measured by dynamic light scattering technique after dilution in deionized water (1: 10 v/v)

[c] measured by electrophoresis technique after dilution in deionized water (1: 10 v/v)

[d] calculated directly as percentage of Sel added, determined by HPLC

[e] calculated directly as percentage of siRNA added, determined by RiboGreen assay

[f] expressed as mean ± SD (n=3)
